# Luminescent quantum dot films improve light use efficiency and crop quality in greenhouse horticulture

**DOI:** 10.3389/fchem.2022.988227

**Published:** 2022-10-20

**Authors:** Damon Hebert, Jeroen Boonekamp, Charles H. Parrish, Karthik Ramasamy, Nikolay S. Makarov, Chloe Castañeda, Lisanne Schuddebeurs, Hunter McDaniel, Matthew R. Bergren

**Affiliations:** ^1^ UbiQD, Inc., Los Alamos, NM, United States; ^2^ Delphy Improvement Centre, Bleiswijk, Netherlands

**Keywords:** quantum dot, nanotechnology, nanomaterials, greenhouse, spectrum, sunlight, horticulture, tomatoes

## Abstract

The spectral quality of sunlight reaching plants remains a path for optimization in greenhouse cultivation. Quantum dots represent a novel, emission-tunable luminescent material for optimizing the sunlight spectrum in greenhouses with minimal intensity loss, ultimately enabling improved light use efficiency of plant growth without requiring electricity. In this study, greenhouse films containing CuInS_2_/ZnS quantum dots were utilized to absorb and convert ultraviolet and blue photons from sunlight to a photoluminescent emission centered at 600 nm. To analyze the effects of the quantum dot film spectrum on plant production, a 25-week tomato trial was conducted in Dutch glass greenhouses. Plants under the quantum dot film experienced a 14% reduction in overall daily light integral, resulting from perpendicular photosynthetically active radiation transmission of 85.3%, mainly due to reflection losses. Despite this reduction in intensity, the modified sunlight spectrum and light diffusion provided by the quantum dot film gave rise to 5.7% improved saleable production yield, nearly identical total fruiting biomass production, 23% higher light use efficiency (g/mol), 10% faster vegetative growth rate, and 36% reduced tomato waste compared to the control, which had no additional films. Based on this result, materials incorporating quantum dots show promise in enabling passive, electricity-free spectrum modification for improving crop production in greenhouse cultivation, but extensive controlled crop studies are needed to further validate their effectiveness.

## Introduction

As the world population continues to increase (United Nations, 2019), and conversely, the arable land per person decreases (United Nations Food and Agriculture Organization, 2018), farmers are faced with the challenge of sustainably producing more fresh food for society in the same amount of space. In addition, food security concerns have underscored the need for local food production, especially in times of crises, when supply chains can be disrupted or are unreliable (National Research Council, 2012; Federoff, 2015). To address these food security issues, efficient farming techniques, such as controlled environment agriculture (CEA), must be adopted and optimized to reliably produce nutritious fruits and vegetables year-round and in diverse climates (Benke and Tomkins, 2017). Greenhouses offer an opportunity to control the growing environment for crops, allowing for reliable harvests and extended growing seasons, while utilizing the energy of the Sun and increasing water use efficiency by over 90% compared to field agriculture (Nederhoff and Stanghellini, 2010).

In the controlled environment of a greenhouse, emerging techniques can be implemented to increase productivity even further, thereby increasing the efficiency of the greenhouse. Until recently, greenhouse technologies focused on the light environment have mainly been developed to achieve the optimal daily light integral (DLI, mol m^−2^ day^−1^) for plants by controlling the photosynthetic photon flux density (PPFD, μmol m^−2^ s^−1^), such as shade cloths or supplemental lighting. While supplemental lighting and shading technology are useful for controlling light intensity and photoperiod, the quality of the spectrum provided to plants remains a parameter to be optimized. Light quality affects both growth (photosynthesis) and development (photomorphogenesis) of plants. Sunlight quality of a greenhouse is a function of the geographical incident solar resource and the transmission properties of the greenhouse façade material. Even in greenhouses with supplemental lighting, the vast majority of photons absorbed by plants are from the Sun, so the solar spectrum typically defines the quality of light the plants receive.

Typical greenhouse glazing materials affect light transmission and/or light diffusion (haze), but do not modify the spectral quality of photosynthetically active radiation (PAR, 400–700 nm) reaching plants. A few spectrum-modifying greenhouse technologies do exist, such as photoselective spectral netting (Shahak et al., 2004), photoselective films (Murakami et al., 1997; Li et al., 2000; Fletcher et al., 2004) or pigmented coatings (Aldaftari et al., 2019; Yalcin and Erturk, 2019) applied to the exterior structure. These technologies rely on filtering out specific wavelength ranges to create a custom light spectrum for plants, but lead to a reduction in total light intensity by as much as 75% (Shahak, 2008). A reduction in light may be suitable for plants that prefer a lower light intensity, but it is not suitable for high-light intensity greenhouse crops such as tomatoes, cucumbers, peppers, or hemp. The reduced light transmission can result in smaller marketable yields and a lower marketable fruit rate (i.e., more waste) (Fletcher et al., 2008). Fruit waste, often a result of deformed or cracked fruit, can affect production as these fruits are not easily marketable and can also provide pathways for disease, insects, and fungi. Many factors have been associated with fruit cracking, but most solutions involve using crack-resistant cultivars, minimizing water stress through proper irrigation, as well as providing more consistent environmental conditions, including the light environment (Khadivi-Khub, 2015). Waste can also be attributed to plants suffering from disease and it has been shown that the presence of even a small portion of UV-B light (280–315 nm) can help control and reduce plant disease in greenhouse crops (Matsuura and Ishikura, 2014; McLay et al., 2020).

One potential solution for modifying the light spectrum in greenhouses is to use luminescent films, which absorb shorter wavelength photons and convert them to longer wavelengths (Pearson et al., 1995; González et al., 2003; Pogreb et al., 2004; Hemming et al., 2005; Hemming et al., 2006; Parrish II et al., 2021). Luminescent films, therefore, differ from filtering technologies as the spectrum can be modified while maintaining higher PAR intensities to achieve higher production yields. Luminescent technologies for greenhouses based on organic dyes or inorganic nanomaterials have been developed. A critical review of extrinsic sensitization strategies through photoluminescent spectral conversion towards ultra-efficient photosynthesis provides a thorough coverage of down-shifting and down-converting materials (Wondraczek et al., 2015). The review highlights the emphasis on the qualitative spectral adjustment that is required to optimize natural photosynthesis. One study demonstrated that spectral conversion, using a Ca/Sr/Eu/S-based photoluminescent phosphor that converts green light to red light to better match the absorption peak of chlorophyll, can improve biomass growth and oxygen production rates in closed-cycle algae reactors (Wondraczek L. B., 2013). One of the earliest examples of a study using phosphor-based sunlight spectral conversion materials over higher plants, in this case *Spinacia oleracea*, showed that a 650-nm emission phosphor conversion foil harvesting green light resulted in an increased CO_2_ assimilation rate by as much as 25% (Xia, et al., 2013).

Additionally, green-to-red converting dyes embedded in greenhouse panels that incorporate traditional solar cells have been developed, which have been shown to generate electricity without reducing growth rates for algae compared to growth under a full solar spectrum (Detweiler et al., 2015). Red dye-infused luminescent solar concentrators have been shown to increase biomass by 26% and phycocyanin production by 44% in algae (Raeisossadati et al., 2019); however, the benefits to plant growth have not been well-established, especially on high-light intensity crops and have focused on producing electricity without affecting crop production.

Quantum dots (QDs) represent suitable inorganic luminescent materials for optimizing the spectrum in greenhouses because they strongly absorb UV light, which is not used for photosynthesis, and a portion of blue light, and emit light towards longer wavelengths that is more photosynthetically efficient for plants. Quantum dots have optimal optical properties due to their high photoluminescence (PL) quantum yield (QY), size-tunable optical properties realized in manufacturing, and inherent photostability compared to organic dyes. Owing to their small size (<10 nm), the absorption profile and peak PL emission of QDs can be tuned during manufacturing by simply changing the size of the nanoparticles.

While there are a variety of QD compositions commercially available, the commercial films used in this study were enabled by CuInS_2_/ZnS (core/shell) QDs. CuInS_2_/ZnS QDs are advantageous over other compositions as they use a low-cost, scalable manufacturing method (McDaniel et al., 2014), have a safer non-toxic composition (contrary to other QD compositions) (Pons et al., 2010; Kubicek-Sutherland et al., 2020), and exhibit strong absorption at wavelengths <400 nm, thus minimizing PAR absorption (see Supplementary Figure S1). Additionally, CuInS_2_/ZnS QDs exhibit high PL QY and also have a wide, size-dependent emission range covering wavelengths between 550–1,300 nm (Chang et al., 2018; Makarov et al., 2019). In a recent study, orange (600 nm) and red (660 nm) CuInS_2_/ZnS QD films were shown to increase edible dry mass (13% and 9%, respectively), edible fresh mass (11% each), and total leaf area (8 and 13%, respectively) in red romaine lettuce (Parrish II et al., 2021). In this paper, large-area CuInS_2_/ZnS QD films were installed inside a greenhouse to passively modify the solar spectrum to improve tomato crop production and reduce fruit waste.

## Results

The objectives of this study are to evaluate the effects of an altered sunlight environment resulting from the application of a retrofit QD film on the growth and fruit production of greenhouse-grown tomato plants. The hypothesis is that a red-shifted spectrum and increased light diffusion compared to a control greenhouse compartment will overcome a reduction in light intensity to result in higher fruit production and increased vegetative growth metrics.

### Quantum dot luminescent films

**FIGURE 1:**
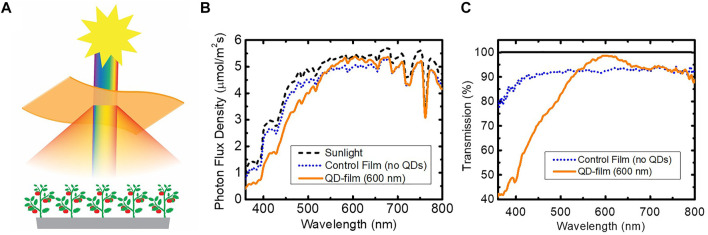
**(A)** Schematic of luminescent QD film technology. Shorter wavelength radiation from the sun is absorbed and down-converted to longer wavelength (lower energy) radiation *via* photoluminescence of a QD fluorophore. The remaining solar spectrum passes through the film to the plants below. The light emitted from the QDs is isotropic, providing a diffuse orange light to the plants below for improved full-canopy light absorption. **(B)** Spectral measurements of sunlight (New Mexico, 25 September 2019, 2:30 p.m., no clouds) and transmitted sunlight spectra through the QD film and a control film which contains no QDs. **(C)** Transmission of incident solar spectrum through the QD film (orange, solid) and through the control film (blue, dotted) compared with incident sunlight.

In this work, luminescent greenhouse films incorporated with CuInS_2_/ZnS (core/shell) QDs were installed in a hydroponic glass greenhouse to study tomato development and production. A schematic is shown in Figure 1A, illustrating that QD films absorb a portion of UV/blue photons from the Sun and emit longer wavelength photons, which are more efficient for photosynthesis. The films are designed to allow some of the UV and blue photons to be transmitted in order to maintain plant health while also increasing the red portion of the solar spectrum. The specific QD film used in this study had a peak emission centered at 600 nm (Supplementary Figure S1).

The transmission properties of the 600 nm-emitting QD film were characterized prior to installation in the greenhouse. Figure 1B shows the incident solar spectrum measured at 2:30p.m. in New Mexico, United States, on 25 September 2019, and the perpendicular transmittance of the solar spectrum through the QD film as well as through a control film, which contained no QDs. While the drop in solar transmittance through the control film is relatively constant over most of the spectral range, mainly due to reflectance, the QD film transmittance showed increased absorption of shorter wavelengths (<550 nm) and QD emission centered at 600 nm (Figure 1C).

The absolute and relative changes in photon flux for the QD film and the control film compared with incident sunlight for different wavebands are shown in Table 1. The total transmission of PAR sunlight through the QD film and the control film was 87.5% and 91.3%, respectively. In Table 1, the “absolute change” indicates the change in photon flux by the QD film for each spectral range compared to incident sunlight. The UV and blue spectral ranges exhibited the most loss due to QD/plastic absorption, while the red spectral range exhibited the least loss due to QD emission. Normalizing by the photon flux density loss, the “relative change” listed in Table 1 indicates the reduction (or increase) for each wavelength range relative to the ratios of spectral components present in the incident solar spectrum. For a given spectral range,

$$Absolute\;change=\Delta {PFD/PFD}_{sun}$$ (1)

$$Relative\;change={\Delta \%PFD/\%PFD}_{sun}$$ (2)

where ΔPFD is the absolute photon flux density (PFD, 350nm–850 nm) loss or gain after passing through the QD film, PFD_sun_ is the PFD of the incident solar radiation, Δ% PFD is the loss or gain of the chosen spectral component as a percentage of total PFD after passing through the QD film, and % PFD_sun_ is the fraction of the chosen spectral component present in the incident solar radiation. The largest reduction in photon flux through the QD film occurred in the UV range (<400 nm) with an absolute reduction of -60.8%, while red light only exhibited a small change in photon flux of -5.0%. A relative spectral boost was measured for green (+6.1%), red (+9.0%), and far-red (+4.4%) compared to the control film, indicating that the QD film provides a spectrum that is weighted towards longer wavelengths, while decreasing UV and blue light.

**TABLE 1:** Absolute and relative percent change of perpendicular transmission of sunlight through the QD film and the control film, compared to incident solar spectrum.

	UV	Blue	Green	Red	Far red
Wavelength (nm)	350–400	400–500	500–600	600–700	700–800
QD Film, absolute change	-60.8%	-29.8%	-7.6%	-5.0%	-9.0%
QD Film, relative change	-55.0%	-19.5%	+6.1%	+9.0%	+4.4%
Control Film, absolute change	-18.9%	-10.3%	-8.4%	-7.8%	-8.0%
Control Film, relative change	-11.1%	-1.7%	+0.4%	+1.0%	+0.9%

Another important optical parameter characterized for the QD film is the hemispherical transmittance, which accounts for the light transmittance over all angles. Measuring the hemispherical transmittance provides a better account for the light distribution in a greenhouse over a day with respect to incident diffuse light, scattered light due to the haze of the film, and the changing solar angle over the day and year. Both the perpendicular (0°) and hemispherical transmittance were measured for the QD film (Supplementary Figure S2; Supplementary Table S1, Supplemental Information), where the perpendicular PAR transmission was measured to be 85.3% while the hemispherical PAR transmission was 79.6%. Similar to the transmission data measured using natural sunlight, the transmission measured at low angles (<45°) and wavelengths >700 nm was constant at 91% and an increase in transmission of 5% centered at 600 nm can be observed in the hemispherical transmission data.

### Tomato plant trial

To investigate the effects of the QD film on crop development, a 25-week tomato trial (March-September 2019) was conducted in glass greenhouses located at the Delphy Improvement Centre in Bleiswijk, Netherlands. The summer of 2019 was characterized by a notorious extreme heat wave in Northern Europe which set all-time temperature records in the Netherlands (39.3°C in North Brabant, a record by 0.3°C) and five other countries. A Dutch orange alert was issued in July for the entire country, and nearly 400 additional people died in the country compared to a regular summer week. This extreme heat made summer cultivation a challenge in Northwest Europe; however, the exposure to heat and light due to weather is an inherent variable in greenhouse production, which is why this study was designed to be conducted in a greenhouse exposed to real-world conditions. It is likely, due to global climate change, that growers will be experiencing more extreme weather similar to 2019 in the near future, which would make these results very relevant for greenhouse cultivation.

**FIGURE 2:**
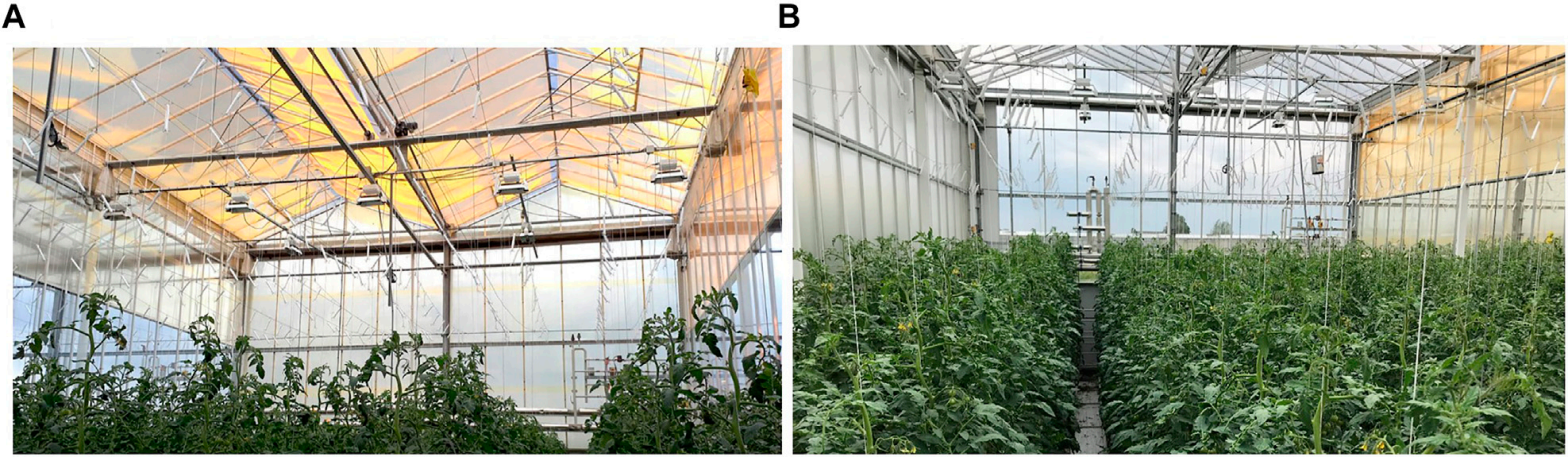
Photographs of **(A)** the test compartment, with QD film installed on the roof, South wall, and top half of East and West walls, and of **(B)** the control compartment with no additional films installed.

The tomato trial consisted of two greenhouse compartments: a test compartment with the QD film installed beneath the greenhouse glass (roof and sidewalls, Figure 2A) and a control compartment which contained no additional films (Figure 2B). The tomato cultivar chosen for the study was a beefsteak variety (*Solanum lycopersicum* L., Merlice). Both the control and test compartments were adjacent, and both were under clear glass, which had no additional light diffusion properties. Measured spectra in both compartments at canopy height are shown in the supplemental information, Supplementary Figure S3.

**FIGURE 3:**
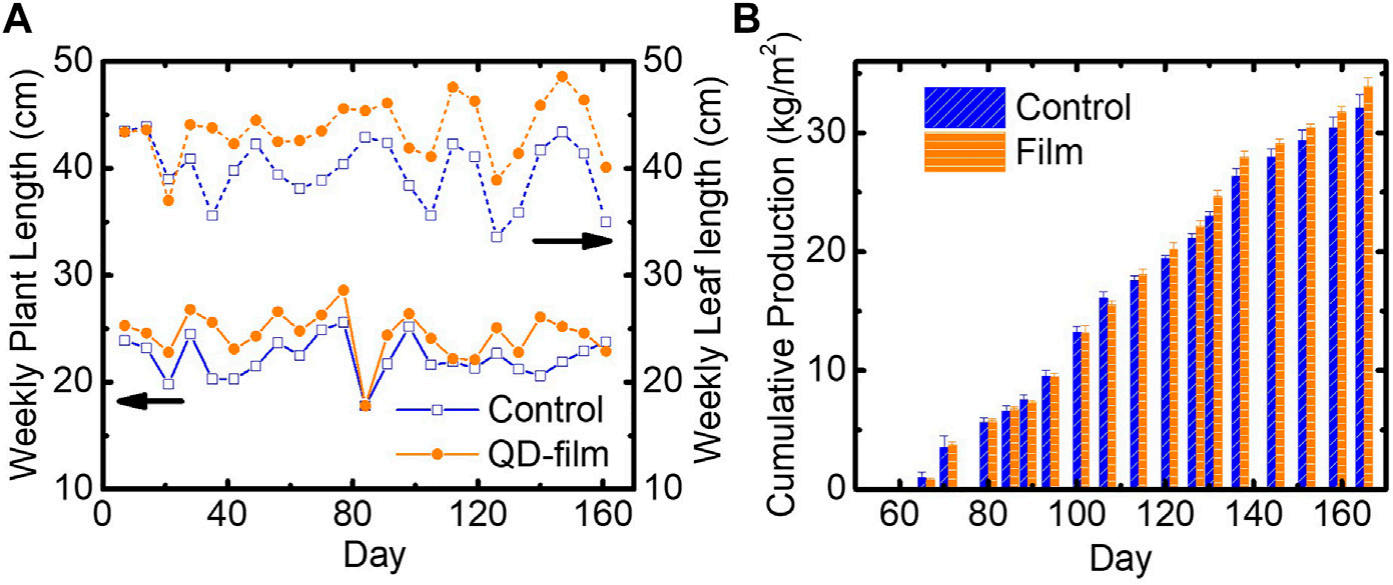
**(A)** Weekly measurements of plant length for plants grown in the control compartment (blue, square, solid) and test compartment (orange, circle, solid) as well as weekly measurements of leaf length for control plants (blue, square, dashed) and plants grown under the QD film (orange, circle, dashed). **(B)** Cumulative salable tomato production under the QD film (orange, horizontal hashed) and under the control environment (blue, diagonal hashed) for the four central gutters.

Throughout the experiment, vegetative growth metrics were monitored by selecting two rows with eight tomato stems in each compartment and the following parameters were measured weekly: vine length, head thickness, number of set trusses, number of flowering trusses, number of fruits set, leaf length, number of leaves, flowering speed, and ripening time (Table 2). On average, plants under the QD film grew 2.1 cm/week faster than control plants, corresponding to a +9.7% increase in vine growth. The average weekly leaf length showed a 3.8-cm increase under the QD film, a +9.5% increase (Figure 3A). Other growth metrics showed negligible differences between the plants grown in the test and control compartments. A *t*-test on two samples assuming unequal variances was performed on vegetative growth metrics using each measurement week for n = 23, resulting in a *p*-values (two tails) as shown in Table 2. *p*-values < 0.05 indicate that the difference in the measured metric was significant over the variance in the dataset. The data show statistical significance for both vine growth and leaf length, but no statistical significance for the other metrics, although production of one additional leaf per plant on average, over the duration of the trial, was relatively close to significant. While internodal spacing was not measured in this study, the observed increase in leaf number, leaf length, and vine length indicates more vigorous growth under the QD film. Flowering speed and fruit set data for the trial can be found in the supplemental information (Supplementary Figure S4).

**TABLE 2:** Growth metrics and relative differences for plants under the QD film and control compartments. *Difference is not statistically significant beyond the variance in the dataset. Metrics that were measured cumulatively for the entire compartment area were not able to be compared for statistical significance, and are marked “N/A”.

Growth metric	Control	QD film	% Change	*p*-value
Vine growth (cm/week)	22.3 ± 1.9	24.4 ± 2.2	+9.7	0.0009
Leaf length (cm)	39.8 ± 3.0	43.6 ± 2.8	+9.5	0.0001
Leaves per plant (#)	30.4 ± 2.1	31.8 ± 2.1	+4.6*	0.1855
Head thickness (mm)	11.7 ± 1.5	11.5 ± 1.3	-2.0*	0.5739
Set trusses (#)	11.2	11.1	-0.5*	N/A
Avg Flowering trusses (#)	11.7	11.6	-1.1*	N/A
Avg Fruits set (#)	5.14 ± 1.66	5.07 ± 1.43	-1.3*	0.8810
Flowering speed (truss #)	0.964 ± 0.157	0.955 ± 0.184	-0.9*	0.8636
Ripening time (days)	59.7 ± 3.6	60.0 ± 2.7	+0.5*	0.7890

Production metrics were monitored after tomatoes had ripened and subsequent fruit harvesting began. The harvested fruits were characterized weekly, and waste fruits were separated from saleable production fruits. Production metrics including cumulative saleable production, average fruit weight, sugar content (Brix), dry matter content, light use efficiency, waste content and total fruiting biomass production (saleable production + waste) and are reported in Table 3.

**TABLE 3:** Production metrics and relative differences for plants under the QD film and control compartments. *Difference is not statistically significant beyond the variance in the dataset. Metrics that were measured cumulatively for the entire compartment area were not able to be compared for statistical significance, and are marked “N/A”.

Production metric	Control	QD film	% Change	*p*-value
Cumulative (saleable) production (kg/m^2^)	32.1 ± 1.1	33.9 ± 0.7	+5.7	0.0419
Average fruit fresh weight (g)	185 ± 17	177 ± 16	-4.6*	0.1432
Average Brix content (°Bx)	4.19 ± 0.28	4.22 ± 0.25	+0.6*	0.9965
Average dry matter content (%)	4.39	4.49	+2.3	N/A
Light use efficiency (g/mol)	7.30	8.95	+22.6	N/A
Cumulative waste (kg/m^2^)	4.1	2.6	-36.0	N/A
Total fruiting biomass, saleable + waste (kg/m^2^)	36.2	36.5	+0.8	N/A

Over the entire trial, saleable production per unit area was improved from 32.1 ± 1.1 kg/m^2^ in the control compartment to 33.9 ± 0.7 kg/m^2^ in the QD film compartment, an overall improvement of +1.8 kg/m^2^, or +5.7%. A *t*-test on two samples assuming unequal variances was performed on cumulative production data using the four central gutters for *n* = 4, resulting in a *p*-value (two tails) of 0.0419, indicating that the yield improvement was significant over the variance in the dataset (*p*-value < 0.05). This saleable production increase is meaningful, given that this increase alone is a large fraction (23–60%) of the entire baseline production (3–8 kg/m^2^) of typical field-grown tomatoes (Nederhoff and Stanghellini, 2010). Figure 3B shows weekly cumulative saleable tomato production of both the control and QD film compartments. Individual weekly production data can be found in Supplementary Figure S5 in the supplemental information.

Tomato plants grown under the QD film produced 2.6 kg/m^2^ of waste fruits (7.1% of total fruit biomass), while plants grown on the control side produced 4.1 kg/m^2^ of waste (11.3% of total fruit biomass), which represents a relative reduction of 36% in fruit waste. Including production and fruit waste together, the two sides of the experiment produced nearly identical total fruiting biomass, as shown in Table 3.

The average fruit size of tomatoes harvested under the QD film was smaller than that of the control (-4.6%), but the difference was close to, but not, statistically significant. Given the larger production yields, this would indicate that more saleable fruit were produced under the QD film; however, the number of fruit set also indicated no statistical difference. The production increase, then, is primarily due to reduced waste content, observed as cracked skin (Figure 5A), under the QD film (see red dashed areas compared in Figure 5B).

Brix measurements (to analyze differences in sugar content) and dry weight were measured bi-weekly for both compartments. A minimal difference in sugar content (Supplementary Figure S6, SI) and a slight improvement in dry weight of +2.3% (Supplementary Figure S7, SI) were recorded, but were not statistically significant.

**FIGURE 4:**
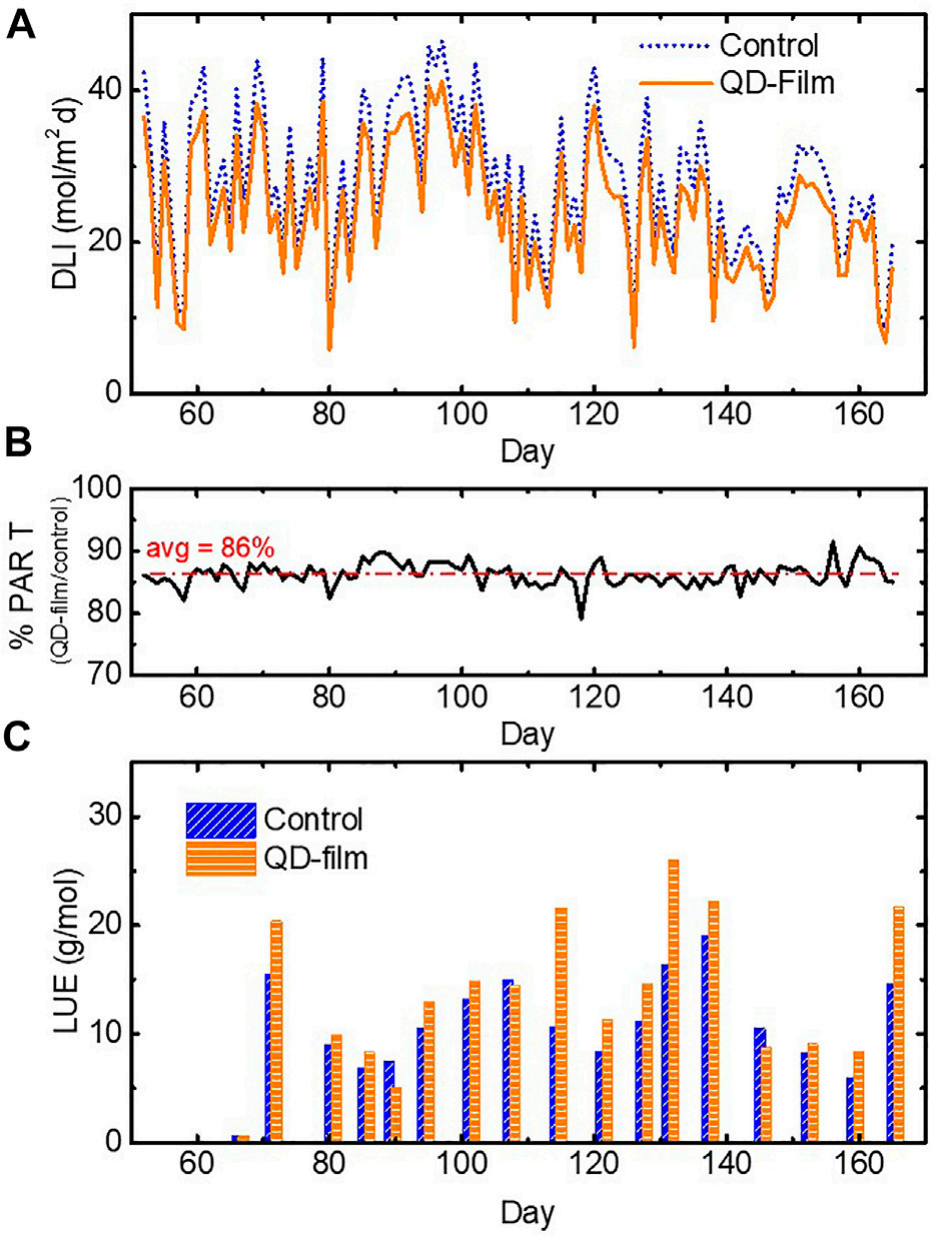
**(A)** Daily light integral recorded under both the control compartment (blue, dotted line) and the QD film compartment (orange, solid line). **(B)** The percentage of weekly PAR transmission under the QD film vs. control with the average PAR ratio in red, dotted. **(C)** Light use efficiency for control (blue, diagonal hashed bars) and QD film (orange, horizontal hashed bars) by harvest.

DLI measurements for both compartments are shown in Figure 4A. The DLI in the control compartment was consistently higher than that in the QD film compartment. Figure 4B shows the daily PPFD deficit in the QD film compartment compared with the control. Over the course of the experiment, the plants under the QD film received 14% lower DLI than did the control plants. Roughly 10% of this loss was due to reflection from the additional two surfaces introduced by the barrier film of the QD film. Normalizing against this DLI reduction leads to a metric known as light use efficiency (LUE), defined as the amount of crop production per unit PAR light (g/mol). Periodic LUE data is shown in Figure 4C. Over the course of the trial, the LUE on in the test compartment was 8.95 g/mol and 7.30 g/mol for the control, an increase of 1.65 g/mol or +22.6%. A *t*-test indicated that the LUE increase was statistically significant.

**FIGURE 5:**
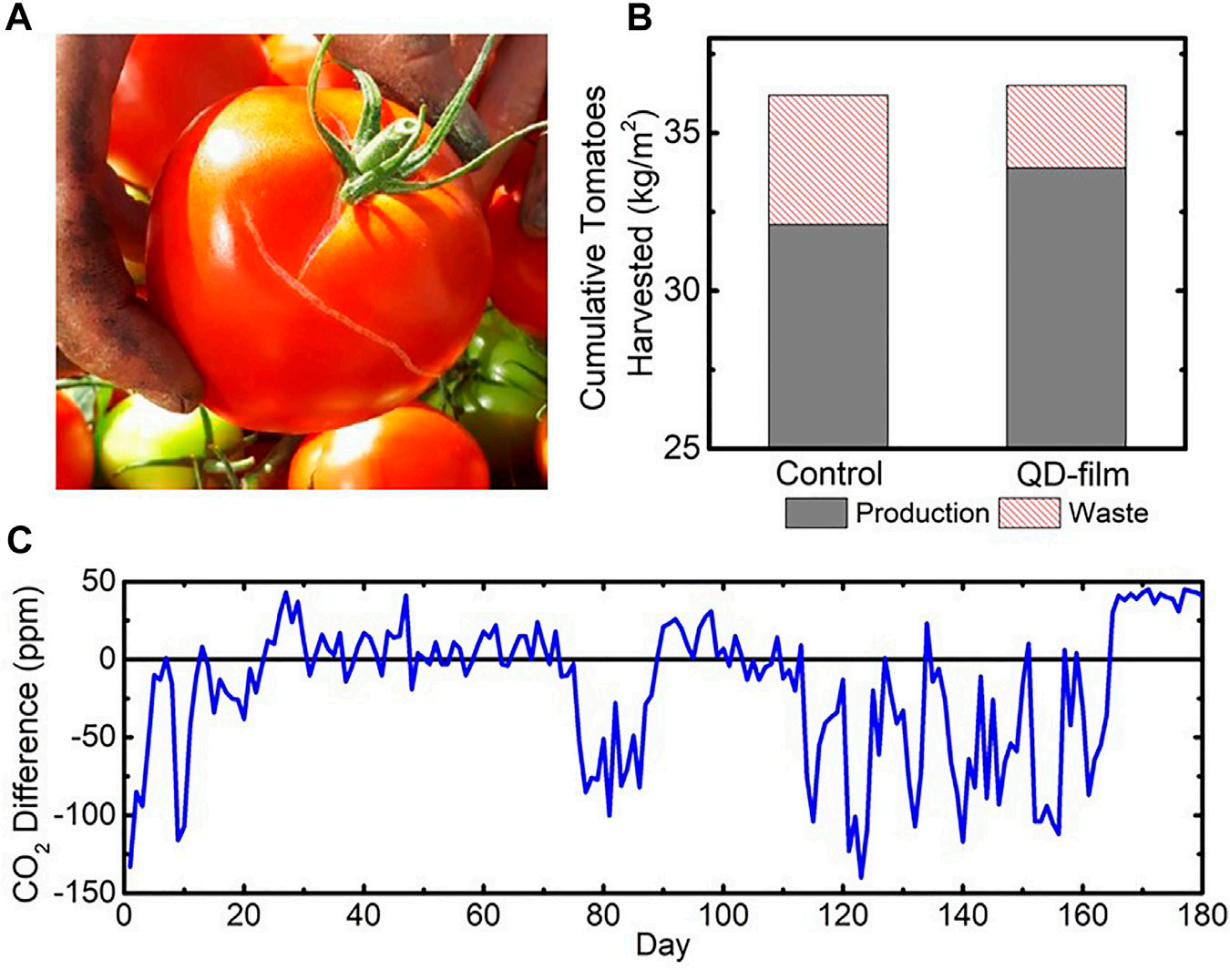
**(A)** Example of a harvested tomato considered “waste” due to cracked skin. **(B)** Comparison of cumulative waste harvested from the control and QD film compartments. **(C)** Difference in CO_2_ concentration between the two compartments (QD film–control) over the course of the trial.

Despite efforts to control all growth parameters between the two compartments, the CO_2_ concentration between control and QD film compartments differed over the course of the experiment (Figure 5C). Installing the QD film in one of the greenhouses resulted in providing extra insulation to the test compartment, compared to the control compartment, which could have caused higher temperatures in the greenhouse containing the QD film. To maintain equal daytime greenhouse air temperatures within an average of 0.2°C, the roof ventilation for the test compartment was opened more frequently to vent hot air. This resulted in an overall reduction in CO_2_ concentration by an average of 21 μmol/mol (ppm) over the trial, from 483 μmol/mol (control) to 462 μmol/mol (QD film). A lower CO_2_ concentration will have an effect on the production of the crop. To estimate how production was potentially impacted under the lower CO_2_ concentration, the following equation was used to approximate the relative increase in photosynthesis due to additional CO_2_ concentration (C, μmol/mol)

$$X=1.5*{\left(1000/C\right)}^{2}$$ (3)

where X is the relative effect of the CO_2_ concentration (in % per 100 μmol/mol) (Nederhoff, 1994). In this case a 462 μmol/mol CO_2_ level would relatively increase photosynthesis by 7.0%, and a 483 μmol/mol CO_2_ level would relatively increase photosynthesis by 6.4%. Therefore, the estimated reduction in production from test compartment due to a lower CO_2_ concentration is ∼0.6%, or equivalently 0.18 kg/m^2^.

## Discussion

QD luminescent greenhouse films, with 600-nm emission, installed over ‘Merlice’ tomato plants in a glass greenhouse compartment, resulted in a more evenly distributed and red-shifted solar spectrum than a comparable control compartment without the QD film. This control compartment and the experimental conditions were designed to unambiguously assign any changes in growth rates and fruit yields to the combined spectral and light distribution alterations offered by the QD film. The choice and the relative orientation of the two compartments, that have historically been used to compare lighting treatments at Delphy Improvement Center, was made such that illumination conditions would be as identical as possible, notwithstanding potential (but undetected) variations that could result from anomalies such as short-term weather, glass transmission properties, reflections from other exterior surfaces, etc.

Despite a 14% reduction in overall DLI under the QD film, this altered spectral and diffuse light environment employed over tomato plants gave rise to a +10% faster vegetative growth rate (vine stem and leaf length) and -36% reduced fruit waste, resulting in an overall +5.7% improved saleable production yield per unit area. With a +4.4% increase in Far-Red light content under the QD film, the increase in vine stem elongation and leaf length are not surprising, especially when accompanied by a reduced DLI.

Normalizing to total DLI, plants grown under the QD film had a 23% higher LUE, indicating an enhanced production efficiency due to light quality and diffusion improvements. Notably, plants under the QD film produced more salable harvest weight per unit area, and nearly identical total fruiting biomass, with a lower DLI, illustrating the effectiveness of the quality of light received under the QD film. Approximately 10% of the DLI reduction was determined to be due to reflection from the film surface itself, indicating that a higher yield improvement could be achieved if QDs could be incorporated into the façade of the greenhouse rather than in the retrofit film tested here.

The production increase also overcame a nonuniformity in CO_2_ concentrations between the two trial compartments. An additional yield improvement of 0.6% for plants grown under the QD film is estimated if the CO_2_ levels were equivalent.

Tomato plants grown under the QD film showed a relative reduction of 36% in fruit waste. In this trial, fruit waste was mainly due to fruit cracking/splitting. While there are many parameters that can contribute to fruit cracking, the three main contributors to skin cracking are due to genetics (Abbott et al., 1986), irrigation stressors, and inhomogeneous climate environment (temperature, humidity, light intensity) (Frazier and Bowers, 1947; Corey and Tan, 1990). Since the same cultivar was used in both compartments, genetics can be ruled out as the main contributor to waste difference. Similarly, there were little differences in irrigation (+2.9% on QD film side) and temperature (0.02 °C warmer on control side) between the two compartments. Therefore, it is postulated that tomato quality difference was likely due either differences in humidity (see Supplementary Figure S8B) between the two compartments, a results of unequal compartment sizes, or the light diffusion benefit resulting from isotropic emission from QDs in the film, offering more uniform light conditions, or more likely a combination of the two. The better light uniformity is thought to reduce hot spots, and improve plant canopy absorption, compared to the control tomatoes grown under clear glass. Diffuse light is known to benefit plant growth by increasing light penetration into the canopy, improving horizontal light distribution, and lowering leaf temperatures, which decreases transpiration (Hemming et al., 2007; Hemming et al., 2008). The QD films act to diffuse direct sunlight in two ways: 1) the luminescence of the QDs is emitted isotropically, and 2) the film itself scatters transmitted sunlight. The total haze of the QD film was measured to be 6.8% ± 0.5%, where 1.8% of the haze is attributed to scattered light and the remaining 5% can be attributed to isotropic emission from the QDs (see Materials and Methods for more information). Due to a more uniform light environment, it is postulated that the transpiration of the crop changed more gradually and that the water balance of the plant was more stable under QD films. Therefore, the fruits could swell gradually, resulting in less tearing of the fruit skin, which occurs when periods of low light are followed by sudden high light intensity conditions.

It is important to note that the improvement in edible fruit production by waste reduction that was observed in this study can be a viable route to improving production for commercial greenhouse growers. The more uniform light conditions along with improved spectral quality by providing additional photons that are more efficient for photosynthesis and achieving better full-canopy absorption of high-quality light all contributed to improved crop development and production. Additionally, more vigorous vegetative growth was observed under the QD film (∼10%). It is possible by modifying the emission of the QDs to create new light recipes or by selecting cultivars with a different source/sink ratio, more of the increased biomass could be directed to the fruits to further improved production.

Future work will include exploring new light recipes and extending the study into the winter season to explore the performance of QD films in a period of lower light level, since many greenhouses operate year-round. Since it was not possible to disentangle the effects related to differences in light diffusion or spectral quality from this study, future experiments should focus on controlling for light diffusion by providing a control greenhouse with a neutral-colored, equally diffusive film that decreases DLI by an equal amount as does the QD film. Furthermore, spectral quality effects on secondary metabolites (Thoma et al., 2020) and shelf life (Lin and Jolliffe, 1996) are potential topics for additional research. Finally, because of the isotropic emission from the QD layer in the current front-face design, some emission is lost in backscattering away from plants, although some of that backscattering is reflected back towards plants from the plastic barrier layer and the greenhouse façade. Further research on engineering of unidirectional light-extracting photonics (Shen, et al., 2021), for example using micropatterning, is needed to improve the efficiency of emitted light that reaches plants.

Overall, this study demonstrated that QD films are a promising technology to improve the sunlight environment in greenhouses that can improve tomato production, light use efficiency and reduce waste production.

## Materials and methods

### QD film measurements

The quantum dot films used for the study are available commercially (UbiGro retrofit greenhouse film, UbiQD, Inc., Los Alamos, United States) and utilize CuInS_2_/ZnS quantum dots incorporated into plastic film. The film was 1.25 m wide, 350 µm thick and the length of the films were cut to fit the dimensions of the greenhouse.

The transmission properties of the QD film were characterized prior to installation in the greenhouse using an Optimum SRI-PL-6000 (Optimum Optoelectronics Corp., Hsinchu, Taiwan) handheld spectrophotometer. Film characterization measurements were made under direct Sun on a cloudless day, on 25 September 2019, at 2:30 p.m. in Los Alamos, NM, United States (2,231 m altitude). Samples were suspended on a level surface 3 cm above the sensor of the spectrophotometer using a 20 cm × 20 cm square aperture.

Both the perpendicular (0°) and hemispherical transmittance were measured by the Wageningen University & Research LightLab using a Transvision Hortiscatter IS-SA under the NEN 2675:2018 standard for the determination of optical properties of greenhouse covering materials and screens (Swinkels, 2012).

The haze of the QD film and the barrier film were characterized following a modified version of the ASTM D1003 standard (ASTM D1003-07, Standard Test Method for Haze and Luminous Transmittance of Transparent Plastics, 2007). The haze measurement system consisted of a near-infrared LED (780 nm), a 12-in diameter integrating sphere, and a fiber-coupled spectrophotometer (Bergren et al., 2018). The NIR LED light source was chosen because it is not absorbed by the QDs, and thus light diffusion due to scattering could be isolated from the isotropic photoluminescence of the QDs. The measured haze due to scattering of the barrier film was 3 ± 2% and that of the QD film was 1.8% ± 0.5%. Haze was also measured using a white light source following ASTM D1003. In contrast to measuring haze with the NIR LED source, by using the white light source, the haze value is affected by a combination of luminescent emission from the QDs and scattering from the barrier films and QD resin. In this case, the haze was 6.8%, indicating that the isotropic emission of the QDs is responsible for the majority of the light diffusion exhibited by the QD film.

### Glass greenhouse plant trial

A 25-week plant trial (March-September 2019) was conducted in glass greenhouses located at the Delphy Improvement Centre in Bleiswijk, Netherlands (52.030591, 4.530305). The plant trial consisted of two greenhouse compartments: a test compartment with the QD film installed beneath the greenhouse glass and a control compartment which contained no additional films. Both the control and test compartments were adjacent, and both were under the same clear glass, which had no additional light diffusion properties. In the test compartment, the South wall, roof, and top 6.7 m of the East and West walls were covered with the QD film. The tilt angle of the greenhouse roof was 21°.

The QD film, in 1.25-wide strips of customized lengths, was installed using plastic clips that fastened the film to the interior metal structure of the greenhouse. The test compartment had a growing area of 112.8 m^2^ which contained six hydroponic gutters to grow *Solanum lycopersicum* L. tomatoes on the vine (TOV). The tomato cultivar chosen for the study was beefsteak variety ‘Merlice’. The TOV were grated on a Maxifort rootstock and topped with two stems per plant. The plants were spaced at a density of 1.8 plants/m^2^, resulting in 3.6 stems/m^2^. The plants were then placed on a stone wool slab for growing. The control compartment also contained six gutters and the same plant density was used, but the growing area was larger (139.2 m^2^). For both compartments, plants grown in edge gutters were designated as buffer areas and were omitted from the study; the central four gutters were used, where light quality was most consistent, away from the greenhouse edge. This reduced the trial growing area to 76.8 m^2^ and 96.0 m^2^ for the test and control compartments, respectively.

The climate of each compartment was managed using a Priva climate computer, with data registration every 5 minutes. Greenhouse temperature, humidity, and irrigation data can be found in Supplementary Figure S8. To achieve the optimal CO_2_ concentrations, pure CO_2_ was dosed in each compartment. Growth parameters other than sunlight spectrum were controlled and all effort was made to achieve equivalence in both greenhouse compartments, but due to size differences of the compartments, it was impossible to balance temperature and humidity in both. Therefore, humidity was higher in the control compartment while temperature was held constant between the two. No supplemental lighting was used in this plant trial. DLI was also measured from two different locations in the center two gutters, sampling every 5 minutes and integrating each day.

Throughout the experiment, vegetative growth metrics were measured as the plants developed, including vine length, head thickness, the number of set and flowering trusses, the number of fruits set, leaf length, number of leaves, flowering speed, and ripening time.

To quantify the tomato production between the control compartment and the QD film compartment, the harvested fruits were measured and analyzed. As fruits were harvested, waste fruit were separated from salable production fruit, and weights were recorded for both groups. Waste fruit were those with obvious blemishes such as skin cracking, bruising or other cosmetic defects. Once harvesting began (at ∼10 weeks), the total production (kg/m^2^) for each compartment was calculated weekly, and the average fruit weight and number of harvested trusses were recorded.

The quality of the fruit was analyzed, where the dry weight from a sample of fruits (5 per measurement) was recorded bi-weekly. The dry weight gives a better indication of the amount of assimilates produced and transported to the fruits. The sugar content (Brix) was also monitored, where the Brix values of ten fruits from each compartment were measured bi-weekly.

## Data Availability

The original contributions presented in the study are included in the article/Supplementary Material, further inquiries can be directed to the corresponding author.
