# Supplementary material for: Luminescent quantum dot films improve light use efficiency and crop quality in greenhouse horticulture
Source: Front Chem. 2022 Oct 20;10:988227. doi: 10.3389/fchem.2022.988227 (PMC9631427; doi:10.3389/fchem.2022.988227)
Supplement: Supplementary file 1 [file DataSheet1.docx]

**Figure S1**. Normalized photoluminescence (orange, right hand axis) and normalized absorption (black, left hand axes) spectra of the CuInS_2_/ZnS quantum dots in toluene used in the quantum dot films.

**Optical Properties**

*Hemispherical Light Transmission*

Hemispherical light transmission was measured at Wageningen University & Research’s light lab, where a 50cm x 50cm sample of the 600-nm emitting QD-film was mounted over a 3’ diameter integrating sphere and a 2000W Xenon lamp was used to illuminate the sample at various angles. The transmitted spectra was then measured at each angle with a spectrometer. Figure S2 illustrates the angular transmittance recorded for angles 0, 15, 30, 40, 45, 50, 60, and 75 degrees and the hemispherical transmittance was calculated from these measurements. Table S1 shows the percent transmittance recorded for each angle.

**Figure S2**. Hemispherical transmittance data for 600 nm emitting QD-films, measured on QD-films at Wageningen University & Research in the Netherlands using the Transvision Hortiscatter IS-SA under the NEN 2675:2018 standard for the determination of optical properties of greenhouse covering materials and screens.

**Table S1.** Angular transmittance for QD-film (600 nm)

| Angle of incidence | 0° | 15° | 30° | 40° | 45° | 50° | 60° | 75° |
| --- | --- | --- | --- | --- | --- | --- | --- | --- |
| Angular transmittance (%) | 87.5 | 87.2 | 86.7 | 84.0 | 85.2 | 83.9 | 78.9 | 55.4 |

**Figure S3:** (a) Photon flux measured at plant canopy height (~4m) for sunlight transmitted through the glass façade and QD-film (Orange, solid line) in the test department and through just glass façade in the control department. (b) Photon flux difference of two spectra shown in (a), with a relative peak at ~600 nm and the majority of absorption in the UV and blue range <500 nm.

**Figure S4**. (a) Flowering speed is a weekly measurement of the highest flowering truss on a vine-type tomato plant; the difference in truss position week-to-week gives the flowering speed in trusses/week. (b) Fruits set is a weekly measurement of the number of newly formed fruits.

**Figure S5**. Weekly production of Merlice tomatoes during 25-week plant trial

**Figure S6**. Brix (sugar) content measured during trial

**Figure S7**. Percent dry matter measured during trial

**Figure S8.** Greenhouse climate data measured during the trial: (a) temperature, (b) relative humidity, (c) humidity deficit, and (d) irrigation usage.
